# Tenofovir to Prevent HIV Infection in Western China: Pragmatic Randomized Controlled Trial

**DOI:** 10.2196/71494

**Published:** 2025-08-20

**Authors:** Yi Tao, Yan Zhang, Bin Peng, Aizhong Zeng, Jianghong Dai, Hao Liang, Juying Zhang, Huarui Shao, Shihan Feng, Xiaoni Zhong, Ailong Huang

**Affiliations:** 1 Phase I Clinical Trial Ward, The First Affiliated Hospital of Chongqing Medical University, Chongqing, China; 2 School of Public Health, Chongqing Medical University, Yixue Rd1#, Yuzhong district, Chongqing, 400016, China, 86 13508312469; 3 The First Affiliated Hospital of Chongqing Medical University, Chongqing, China; 4 School of Public Health, Xinjiang Medical University, Wulumuqi, China; 5 School of Public Health, Guangxi Medical University, Nanning, China; 6 West China School of Public Health, Sichuan University, Chengdu, China; 7 Department of Pharmacy, Bishan Hospital of Chongqing Medical University, Yixue Rd 1, Yuzhong DistrictChongqing, China

**Keywords:** pre-exposure prophylaxis, HIV, tenofovir, medication compliance, prep regimen, MSM

## Abstract

**Background:**

Pre-exposure prophylaxis (PrEP) programs have been implemented in multiple countries. Evidence from clinical trials and cohort studies has proven the safety and effectiveness of PrEP. However, minimizing drug-related adverse effects and cost should be primarily considered in PrEP. Most trials used tenofovir combined with emtricitabine as the intervention; yet, the use of tenofovir disoproxil fumarate (TDF) (ie, Tenofovir) alone has not been thoroughly evaluated. Furthermore, the medication regimen in most trials was used every day, with a few studies proposing an optimal medication regimen for PrEP.

**Objective:**

This study was designed to systematically evaluate the preventive efficacy and safety profile of TDF-based PrEP in the Chinese population. We also aimed to explore medication compliance, changes in sexual behavior, and hazard factors of HIV infection.

**Methods:**

We conducted a pragmatic randomized controlled trial (RCT) to evaluate the effectiveness and safety of TDF for HIV PrEP. Participants were randomly assigned (1:1:1) to a time-driven group (TDF 300 mg administered orally once daily), an event-driven group (TDF 300 mg administered orally 24 to 48 h before sexual activity and 2 hours after sexual activity, not exceeding 300 mg within 24 h), or an untreated control group. The primary outcomes were the effectiveness and safety of TDF during periods of PrEP use. Secondary outcomes focused on the effectiveness of TDF among participants with good compliance during PrEP use. Tertiary outcomes included the risk factors of HIV infection and behavioral changes from PrEP initiation to the last visit. For ethical reasons, all participants received condoms and health education. This study was registered with the Chinese Clinical Trial Registry (ChiCTR-TRC-13003849).

**Results:**

A total of 1914 participants underwent randomization. During the follow-up of 3513.5 person-years from June 2013 to May 2016, HIV seroconversion was observed in 30 persons (2.02 per 100 person-years) in the time-driven group (time-driven vs control group: hazard ratio [HR] 0.93, 95% CI 058‐1.51; *P*=.78), 35 (1.73 per 100 person-years) in the event-driven group (event-driven group vs control group: HR 0.83, 95% CI 0.52‐1.31; *P*=.42), and 37 (2.06 per 100 person-years) in the control group. Post hoc analysis showed that participants with good medication compliance reduced their HIV infection risk by 53% (*P*=.01) and event-driven medication with good compliance reduced the risk by 62% (*P*=.009). We recorded no severe adverse events during the trial. For tertiary outcomes, low medication compliance, sexual role, no condom use, and more number of sexual partners remained significantly associated with HIV risk.

**Conclusions:**

The TDF-based PrEP is ineffective without good adherence. However, when medication compliance is achieved, event-driven dosing is recommended as an optimal PrEP regimen.

## Introduction

While the global AIDS epidemic remains a persistent public health threat, advancements in antiretroviral therapy and prevention strategies have substantially reduced mortality and improved treatment access, albeit unevenly across regions and populations. In 2023, there were approximately 1.3 million new HIV infections and 630,000 AIDS-related deaths globally, reflecting reductions in both new infections and mortality compared to previous years [1]. HIV remains a major public health issue in China, with an estimated 570,576 HIV infections between 2005 and 2020, particularly concentrated in western China [2]. In 2023, data released by China’s National Administration of Disease Control and Prevention and the Chinese Center for Disease Control and Prevention (China CDC) provided a clear overview of the HIV/AIDS epidemic in mainland China. Notably, the provinces of Sichuan, Guangxi, and Chongqing reported the highest HIV infection rates nationwide. Additionally, regions such as Xinjiang, Yunnan, and Guizhou also emerged as high-prevalence areas, with the epidemic primarily concentrated in western China [3]. In 2023, men who have sex with men (MSM), bisexual individuals, sex workers, and people who inject drugs remained the highest-risk groups for HIV, with sexual transmission accounting for more than 95% of new infections in China [1,4]. MSM continue to represent the group with the highest HIV incidence rate globally, with infection rates rising nearly 15-fold from 1.5% in 2006 to 25.6% in 2022 [5,6].

In the absence of an effective vaccine, core strategies for AIDS prevention include HIV testing [7], circumcision [8], condom use, and health education [9]. However, these measures alone are insufficient. The introduction of tenofovir-based pre-exposure prophylaxis (PrEP) has revolutionized HIV prevention globally [10]. PrEP involves taking antiretroviral drugs before potential exposure to HIV. It is now widely available as a biomedical prevention method to reduce the risk of HIV infection. The coformulation of two nucleoside or nucleotide reverse transcriptase inhibitors has proven highly effective as PrEP in various populations worldwide [11]. The compound tablet emtricitabine (ETC)/tenofovir disoproxil fumarate TDF (Truvada) was licensed by the US Food and Drug Administration in 2012 as the first HIV PrEP drug, with studies showing it can reduce HIV incidence by 44% [12]. However, PrEP’s effectiveness is closely linked to adherence [13]. A notable study has demonstrated that cisgender women consistently adhering to a daily or high-adherence regimen of PrEP using ETC and TDF experienced significantly low HIV incidence [14]. Regarding the optimal dosing regimen, recent research suggests no significant difference in efficacy between daily and event-driven PrEP regimens [15,16]. A study conducted in sub-Saharan Africa indicated that event-driven PrEP regimen provided protection across foreskin tissue, highlighting the need for further clinical evaluation of precoital PrEP, specifically for insertive sex [16].

Despite substantial investments in HIV prevention over the past decades, this field continues to struggle with inadequate long-term investment strategies and resource disparities [17]. PrEP has been implemented as a national health program in multiple countries [18]. Acknowledging its substantial cost, ensuring cost-effectiveness and demonstrating potential efficacy are paramount considerations in the implementation of PrEP [19-21]. The majority of PrEP studies have focused on coformulation of two nucleoside or nucleotide reverse transcriptase inhibitors [12,21-23]. Nevertheless, the exploration of PrEP, particularly the use of TDF monotherapy, remains inconclusive. TDF alone is associated with a lower cost, making it a more accessible option in resource-limited settings where budget constraints are critical [24]. Additionally, by using TDF alone, the regimen may potentially reduce the incidence of certain side effects such as gastrointestinal discomfort or other FTC-related adverse reactions, which could improve patient adherence and overall quality of life [23]. If TDF is proven to be both effective and safe, it should be regarded as a pivotal public health intervention.

We designed a randomized controlled trial in western China with the primary objective of assessing the effectiveness and safety of TDF use alone among MSM. Furthermore, we aimed to gain insights into its efficacy and safety when administered with high compliance rates. Additionally, we aimed to delve into identifying the optimal medication regimen, hazard factors contributing to HIV infection, as well as examining changes in sexual behavior patterns.

## Methods

### Study Design

This trial was a multiprovince, open-label, randomized controlled trial conducted in western China, provinces including Chongqing, SiChuan, GuangXi, and XinJiang.

### Participants

A total of 1500 participants were planned for recruitment at four sites, including Chongqing, Guangxi, Xinjiang, and Sichuan, using a combination of competitive enrollment and snowball sampling methods. By leveraging existing cohorts in each province, researchers streamlined the recruitment process. Eligible participants were MSM aged 18 to 65 years, who reported engaging in sexual activity at least once every two weeks on average and having had at least one sexual encounter with a same-sex partner in the month preceding the trial. Exclusion criteria included participants who were HIV-, HBV-, or HCV-positive; had severe underlying medical conditions; had a history of alcoholism or drug dependence within the past year prior to screening; or had a history of severe allergies.

### Ethical Considerations

This study was conducted in accordance with the principles of the Declaration of Helsinki and ICH-GCP (International Council for Harmonisation Good Clinical Practice guidelines). All volunteers provided written informed consent prior to the start of the study. Participants were informed that study data would be deidentified and their participation was voluntary. They were assured that their information would be used solely for this study, and their questionnaire responses would not affect their careers. The study covered the medical expenses of any drug-related adverse reactions or adverse events. No additional financial compensation was provided to participants beyond the standard study procedures. However, participants assigned to the intervention group received free TDF medication, and all participants had access to free condoms and comprehensive health education. In case of irreversible damage, corresponding economic compensation was provided in accordance with national laws. The trial protocol was approved by the Ethics Committee of Chongqing Medical University and registered with the Chinese Clinical Trial Registry (ChiCTR-TRC-13003849).

### Randomization and Masking

Randomization was performed using a central, computerized system using block randomization, stratified by hospital. Except for questionnaire administrators, neither patients nor other researchers were blinded to group allocation. Participants were randomly assigned in a 1:1:1 ratio to the time-driven group (TDF 300 mg administered orally once daily), the event-driven group (Tenofovir 300 mg administered orally 24‐48 h before and 2 h after sexual activity, not exceeding 300 mg within 24 h), or the untreated control group.

### Procedures

Baseline information encompassing demographic data, medical histories, and sexual behavior patterns was meticulously documented (see Multimedia Appendix 1). Comprehensive laboratory assessments, including tests for transfusion-associated infections such as HIV, HBV, and HCV, along with electrocardiograms, were conducted, with all participants undergoing an HIV test on the day of randomization. Interventions were tailored to the standard operating procedures at each study site. Follow-up visits were scheduled at regular intervals of 12 weeks, extending up to 96 weeks, and encompassed a range of evaluations. These included repeat HIV testing, routine laboratory investigations, assessments of medication adherence, inquiries into sexual behavior and sexually transmitted infections (STIs), collection of medication history, and comprehensive physical examinations. Adherence to these follow-up visits was mandatory for all participants.

### Outcomes

The primary outcomes were effectiveness and safety of TDF during individuals’ periods of PrEP use. Secondary outcomes assessed the effectiveness of TDF for participants with good compliance during periods of PrEP use. Tertiary outcomes included the risk factors of HIV infection and behavioral change from PrEP initiation to the last visit.

### Statistical Analysis

Categorical data were presented as numbers (frequencies) and analyzed using 2-tailed *χ*^2^ tests or Fisher exact test. Continuous variables were presented as means and SD for normally distributed data and median and IQR for skewed data. Groups were compared using one-way ANOVA or the Kruskal-Wallis test. Overall incidence rates per 100 person-years during the follow-up period were calculated for all adverse drug reactions. The risk of HIV infection was analyzed using Cox proportional hazards models, with hazard ratios, corresponding 95% CIs, and associated *P* values.

The ‘survcutpoint’ function from the R package *survminer* was used to determine the cutoff value for medication compliance, which was estimated based on pill counts and self-reports. The optimal critical value for good medication compliance was established by combining data from previous studies [23]. The Kaplan-Meier method and log-rank tests were used to analyze the incidences of HIV seroconversion and medication compliance rates among the groups. The multiple imputation method was used to address missing data on the number of sexual partners, sexual acts, and condom use, with five imputations conducted.

Statistical analyses were conducted using R software (version 4.2.1; Foundation for Statistical Computing) and Stata software (version 17; StataCorp). A two-sided *P* value <.05 was considered statistically significant. For comparison between the three groups, the significance level was adjusted using Bonferroni adjustment.

## Results

**Figure 1.**
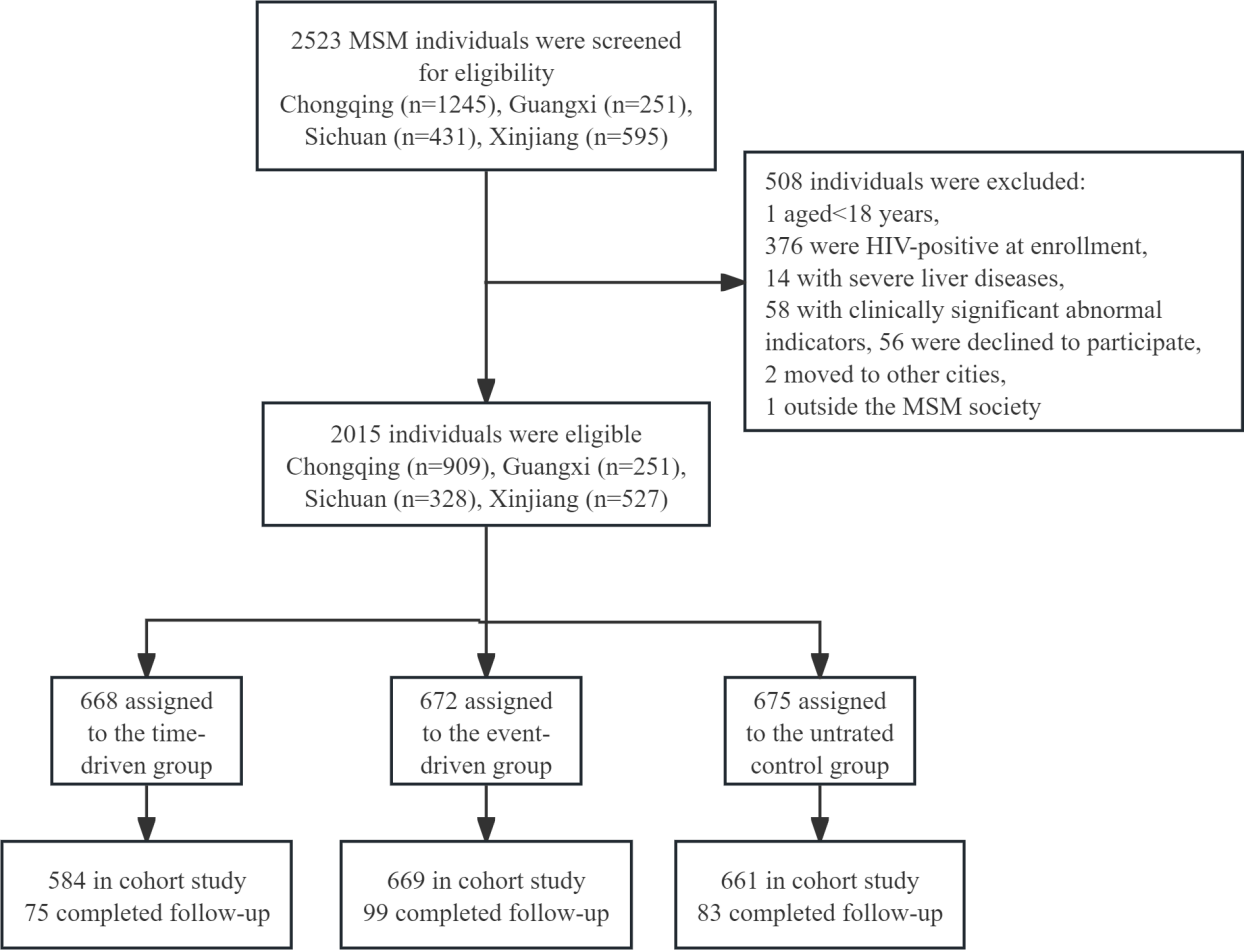
Participants flow chart. MSM: men who have sex with other men.

From June 2013 to May 2016, 2015 participants were enrolled across four sites: Chongqing (n=909), Guangxi (n=251), Xinjiang (n=328), and Sichuan (n=527), among whom 1914 individuals took at least one medication and participated in the study. This cohort was subsequently followed for a total of 3513.5 person-years, with a median follow-up period of 1.5 years per participant (Figure 1). Baseline characteristics were balanced among the three groups: time-driven (n=584; mean 29, SD 8.4 years), event-driven (n=669; mean age of 30 SD 8.4 years), and untreated control group (n=661; mean age of 30.5 SD 9.2 y) (Table 1).

**Table 1.** Baseline characteristics of participants (N=1914).

Characteristics	Time-driven group (n=584)	Event-driven group (n=669)	Untreated control group (n=661)
Age (years), median (IQR)	29.02 (8.43)	29.98 (8.38)	30.48 (9.23)
Nationality (n=1913), n (%)			
Han	541 (92.80)	617 (92.23)	619 (93.65)
Uygur	3 (0.51)	7 (1.05)	6 (0.91)
Hui	7 (1.20)	10 (1.49)	11 (1.66)
Other	32 (5.49)	35 (5.49)	25 (3.78)
Degree of education (n=1912), n (%)^a^			
High school and below	225 (38.53)	239 (35.72)	288 (43.57)
Colleges	142 (24.36)	155 (23.20)	168 (25.42)
Bachelor’s degree or above	216 (37.05)	274 (41.02)	205 (31.01)
Registered residence (n=1908), n (%)			
Urban	415 (71.31)	497 (74.40)	456 (69.30)
Rural	167 (28.69)	171 (25.60)	202 (30.70)
Marriage, n (%)^a^			
Unmarried	458 (78.42)	491 (73.39)	478 (72.31)
Married	87 (14.90)	110 (16.44)	130 (19.67)
Other	39 (6.68)	68 (10.16)	53 (8.02)
Occupation (n=1906)			
Public institutions and enterprises	65 (11.21)	86 (12.89)	73 (11.08)
Professional work	128 (22.07)	182 (27.29)	142 (21.55)
Physical work	30 (5.17)	43 (6.45)	49 (7.44)
Commercial work	82 (14.14)	89 (13.34)	86 (13.05)
Service Industry	89 (15.34)	79 (11.84)	95 (14.42)
Farmers and herdsmen	3 (0.52)	2 (0.30)	3 (0.46)
Students	91 (15.58)	94 (14.05)	83 (12.59)
Unemployed	54 (9.25)	49 (7.32)	76 (11.53)
Other	38 (6.51)	43 (6.43)	52 (7.89)
Monthly disposable income (Chinese Yuan)			
≤3000	309 (52.91)	326 (48.73)	366 (56.22)
3000‐5000	192 (32.88)	248 (37.07)	212 (32.57)
≥5000	75 (12.84)	88 (13.15)	73 (11.21)

a Statistical differences among the three groups.

### Primary Outcomes

**Figure 2.**
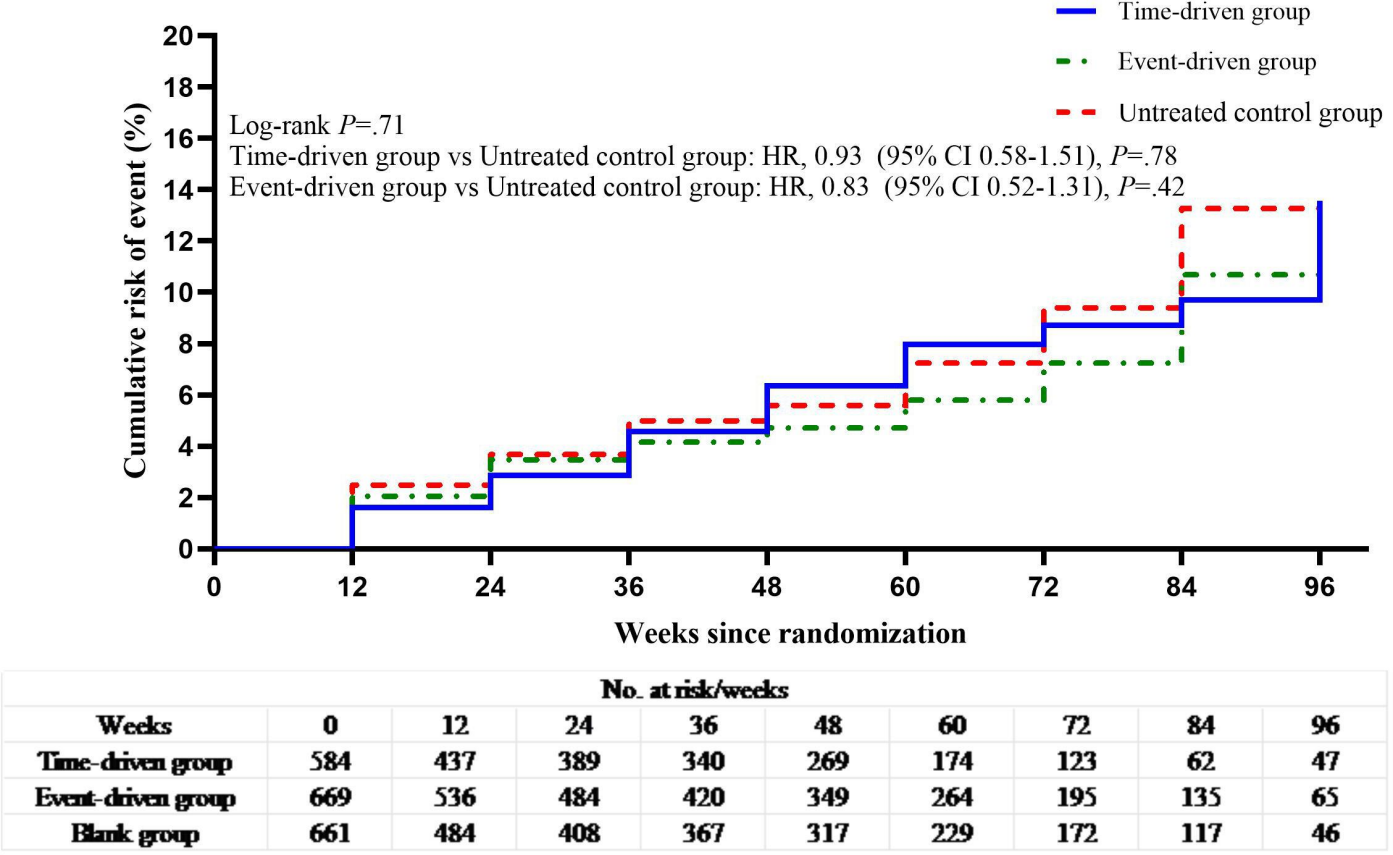
Kaplan-Meier estimates of HIV seroconversion in the time-driven, event-driven, and untreated control groups.

HIV seroconversion was observed in 30 participants (incidence rate [IR] 5.1%, 2.02 per 100 person-years) in the time-driven group (time-driven group vs untreated control group, hazard ratio [HR] 0.93; 95% CI, 0.58‐1.51, *P*=.78), 35 participants in the event-driven group (IR 5.2%, 1.73 per 100 person-years) (event-driven group vs untreated control group: HR 0.83; 95% CI, 0.52‐1.31, *P*=.42) and 37 participants in the untreated control group (IR 5.6%, 2.06 per 100 person-years) (Figure 2).

**Figure 3.**
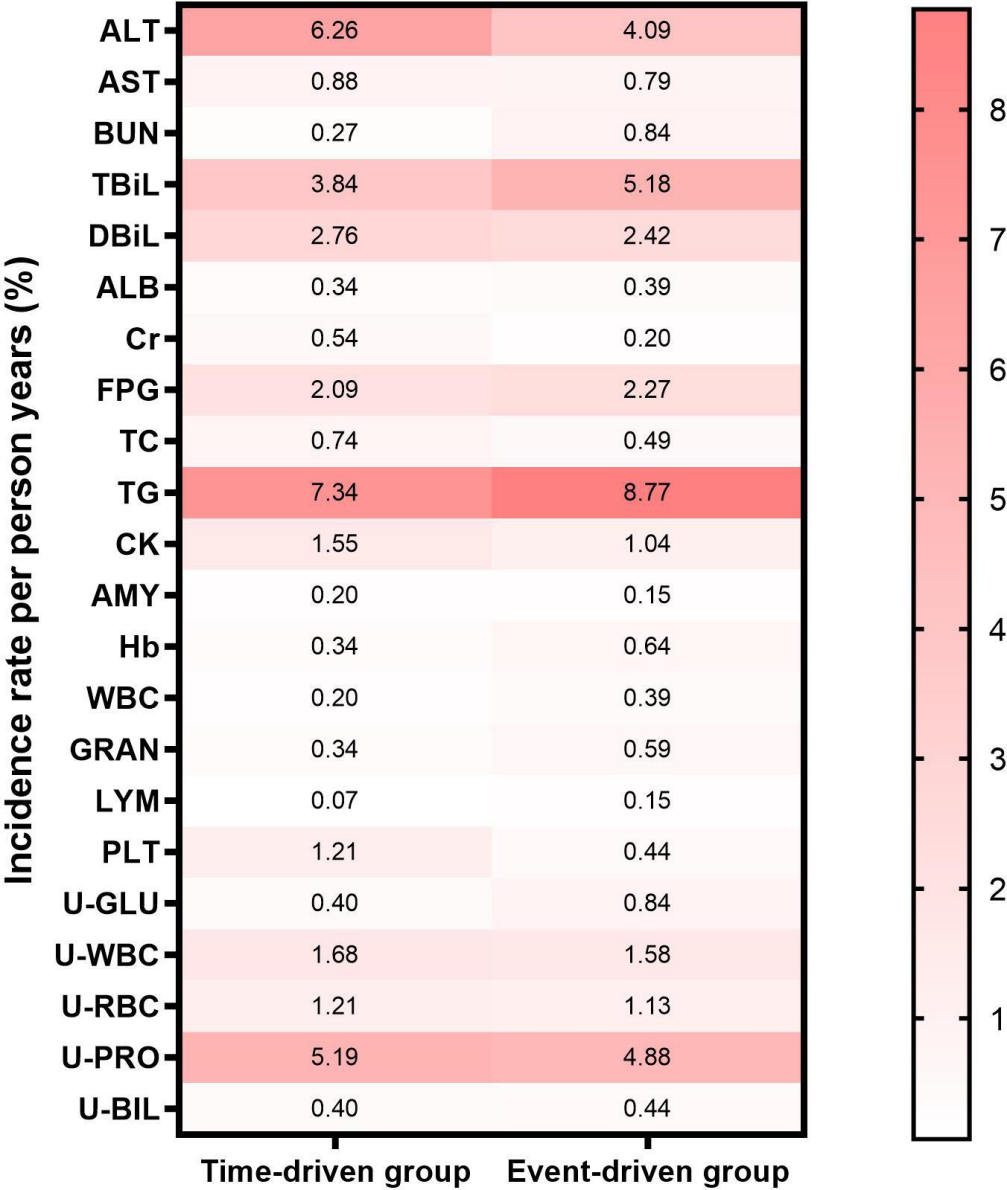
Adverse events in time-driven group and event-driven groups. ALT: Alanine Aminotransferase; AST: Aspartate Aminotransferase; BUN: Blood Urea Nitrogen; TBiL: Total Bilirubin; DBiL: Direct Bilirubin (also known as Conjugated Bilirubin); ALB: Albumin; Cr: Creatinine; FPG: Fasting Plasma Glucose; TC: Total Cholesterol; CK: Creatine Kinase; AMY: Amylase; Hb: Hemoglobin; WBC: White Blood Cell Count; GRAN: Granulocyte Percentage; LYM: Lymphocyte Percentage; PLT: Platelet Count; U-GLU: Urine Glucose; U-WBC: Urine White Blood Cells; U-RBC: Urine Red Blood Cells; U-PRO: Urine Protein; U-BIL: Urine Bilirubin.

No severe adverse events were reported. For dosing as a precautionary medication, laboratory test indicators exceeding 1.5 to 2 times the upper limit of the normal range were regarded as abnormal. The incidence of composite drug-related adverse events was 39.33%. Rates of abnormality in laboratory indicators in the time-driven and event-driven groups were 37.9% and 37.7%, respectively. The toped three abnormalities were elevated triglycerides (8.2%), alanine aminotransferase (ALT; 5.0%), and urine protein (5.0%). Symptomatic adverse events were reported in 1.5% and 1.6% of participants, respectively, with the top-ranked three abnormalities being diarrhea, vomiting, and nausea (Figure 3).

The cohort was followed for 3513.5 person-years with a variable duration of visits (median 1.5 y). HIV seroconversion was observed in 30 participants (HR, 5.1%, 2.02 per 100 person-years) in the time-driven group (time-driven group vs blank group, HR, 0.93; 95% CI, 0.58‐1.51, *P*=.78), 35 (5.2%, 1.73 per 100 person-years) in the event-driven group (event-driven group vs blank group, hazard ratio, 0.83; 95% CI, 0.52‐1.31, *P*=.420) and 37 (5.6%, 2.06 per 100 person-years) in the blank group.

### Secondary Outcome

**Figure 4.**
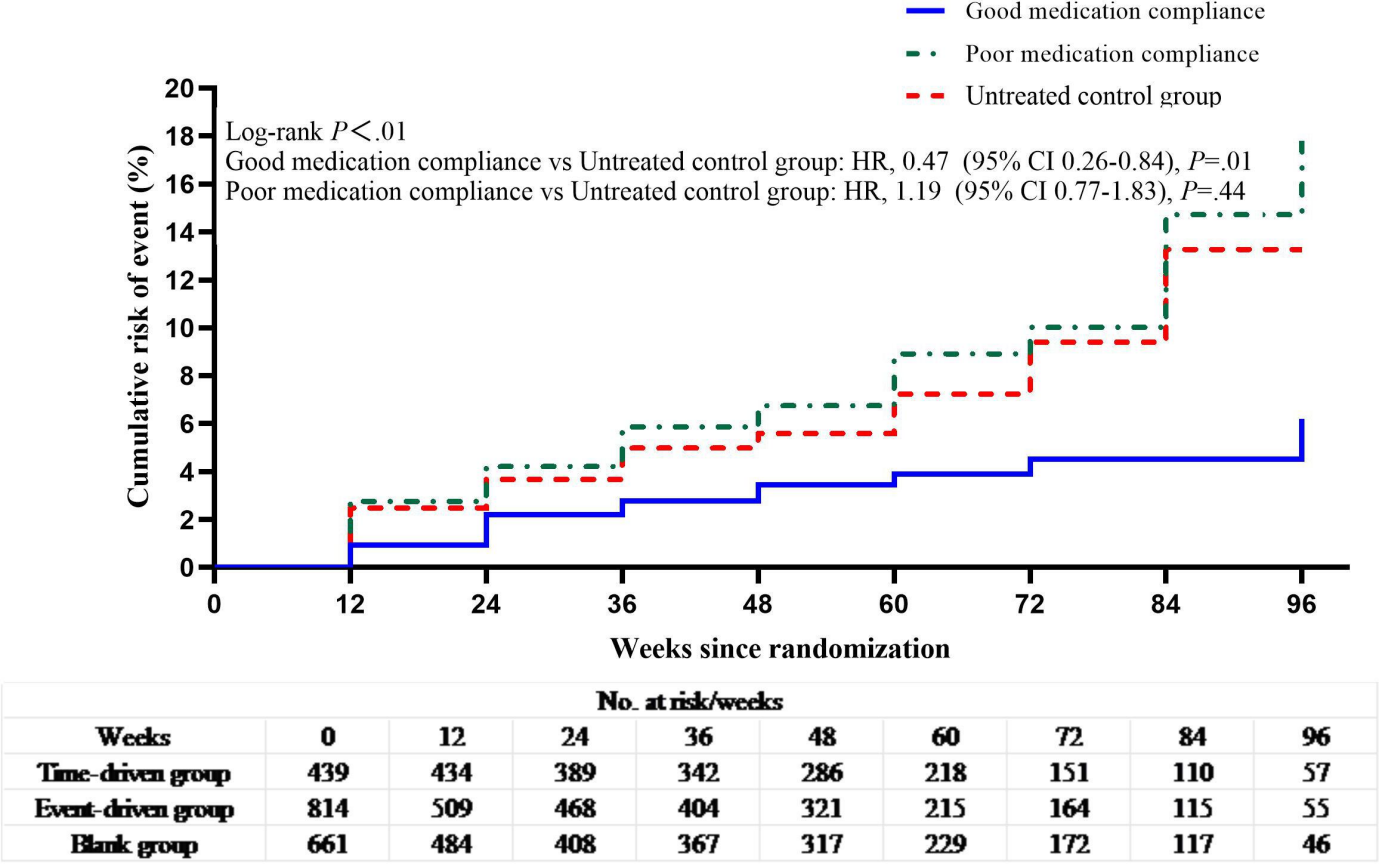
Kaplan-Meier estimates of HIV seroconversion for good medication compliance, poor compliance, and untreated control.

**Figure 5.**
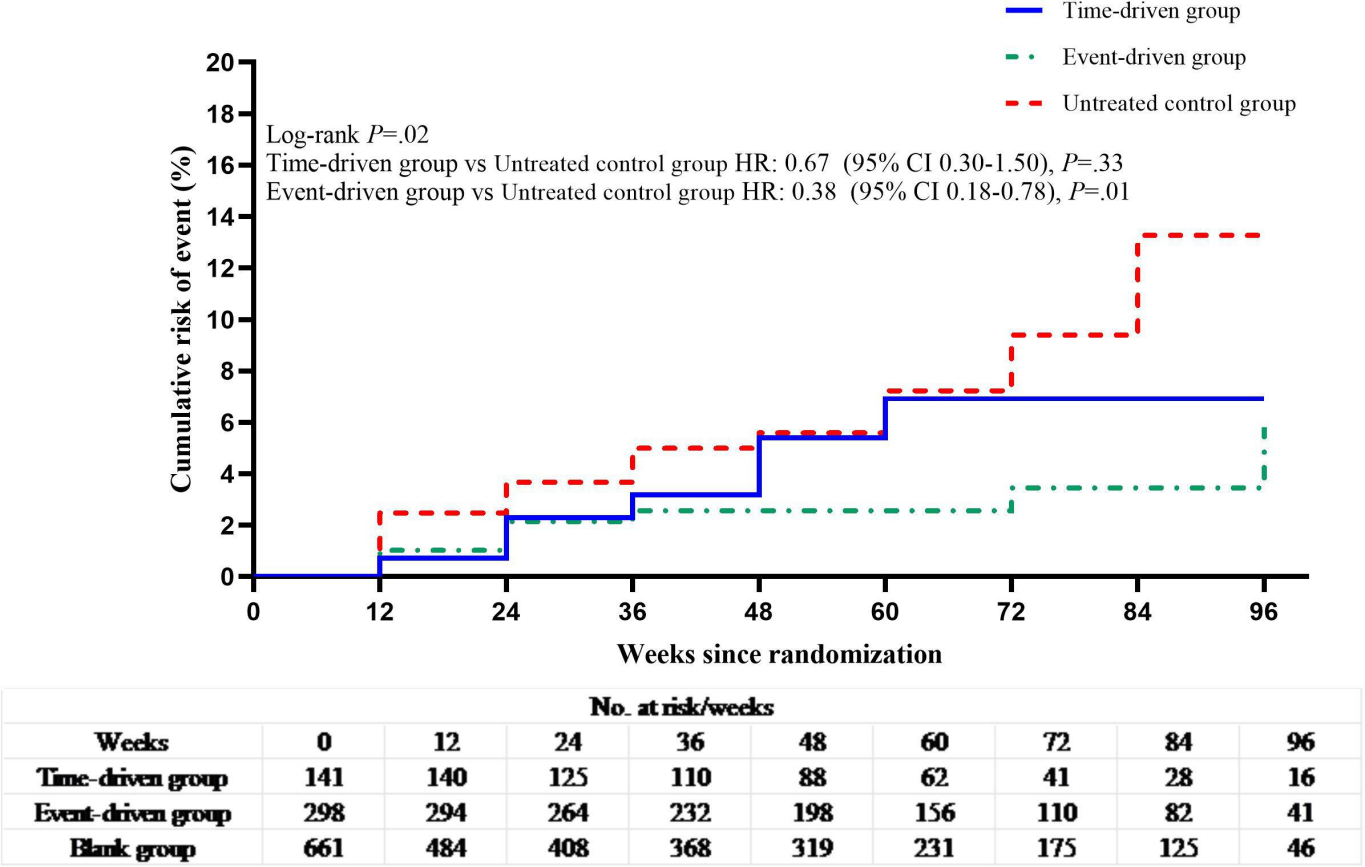
Kaplan-Meier estimates for HIV seroconversion in time-driven group, event-driven group, and untreated control groups with medication compliance rate ≥80%.

The overall average medication compliance rate was 68.4%; event-driven group (77.0%) revealed higher adherence than in the time-event group (57.4%). The cutoff value calculated by *R* software was 40%, double the cutoff value and integrated with previous studies, we defined the optimal critical value of good medication compliance as 80% [25]. Kaplan-Meier estimates showed that participants with good medication compliance had a 53% reduced hazard of HIV infection compared with the untreated group (*P=*.01) (Figure 4). Additionally, time-driven PrEP reduced HIV infection hazard by 62% among those with ≥80% compliance (*P＜*.01) (Figure 5).

After adjustment for medication compliance rate ≥ 80%, good medication compliance reduced the hazard of HIV infection by 53% (*P*=.01).

Similarly, event-driven dosing reduced the hazard by 62% (*P*=.01).

### Tertiary Outcome

Both unadjusted and adjusted HRs for factors associated with HIV infection during follow-up are shown in Table 2. Bivariable Cox regression analyses identified several characteristics significantly associated with HIV infection, including medication compliance, residence, sex role, condom use, number of sexual partners, casual sexual behavior, STI diagnosis, commercial sex, and drug addiction. In the multivariable model, medication compliance, sex role, condom use, and number of sex partners remained significantly associated with HIV risk (Table 2).

**Table 2.** Unadjusted and adjusted hazard ratios for factors associated with HIV diagnosis among participants during follow-up n=1914. Note: 1. Sex role, “0” represents a receptive/bottom role; “1” represents a insertive/top role; “0.5” represents a versatile/switch role. 2. “Drug” refers to recreational substances (eg, alcohol, cannabis, ecstasy, poppers).

Characteristics	Number of HIV seroconversion (%)	Bivariable analysis		Multivariable analysis	
Unadjusted hazard ratio (95% CI)	*P* value	Adjusted hazard ratio (95% CI)	*P* value
Group					
Untreated control group (n=661)	37 (5.60)	1 (Reference)	—^b^	—	—
Time-driven group (n=584)	30 (5.14)	0.93 (0.58‐1.51)	.78	—	—
Event-driven group (n=669)	35 (5.23)	0.83 (0.52‐1.31)	.42	—	—
Dosing			.51		—
No (n=661)	37 (5.60)	1 (Reference)			
Yes (n=1253)	65 (5.19)	0.87 (0.58‐1.31)			
PrEP compliance			.001	—	.001
≥80% (n=434)	16 (3.69)	1 (Reference)		1 (Reference)	
<80% (n=509)	49 (9.63)	2.55 (1.44‐4.51)		2.97 (1.59‐5.54)	
Age	—	0.98 (0.95‐1.00)	.07	—	—
Residence	—	—	.04	—	.07
Urban (n=1368)	65 (4.75)	1 (Reference)		1 (Reference)	
Rural (n=540)	37 (6.85)	0.66 (0.44‐0.98)		1.67 (0.95‐2.93)	
Education					
High school and below (n=236)	14 (5.93)	1 (Reference)		—	—
College degree (n=981)	60 (6.12)	1.07 (0.60‐1.92)	.82	—	—
Bachelor’s and above (n=695)	28 (4.03)	0.71 (0.37‐1.35)	.30	—	—
Career					
Civil service institutions (n=109)	4 (3.67)	1 (Reference)	—	—	—
Corporation (n=824)	39 (4.73)	0.53 (0.18‐1.56)	.25	—	—
Service industry (n=263)	18 (6.84)	0.68 (0.39‐1.19)	.18	—	—
Physical labor (n=263)	18 (6.84)	0.96 (0.50‐1.85)	.91	—	—
Marriage			.77	—	—
Unmarried (n=1587)	83 (5.23)	1 (Reference)			
Married (n=327)	19 (5.81)	1.04 (0.78‐1.40)			
Monthly income					
≤3000 (n=1001)	58 (5.79)	1 (Reference)	—	—	—
3000-5000 (n=652)	34 (5.21)	0.95 (0.63‐1.46)	.83	—	—
≥5000 (n=236)	9 (3.81)	0.79 (0.39‐1.60)	.51	—	—
Sexual roles					
1 (n=923)	43 (4.66)	1 (Reference)	—	1 (Reference)	—
0 (n=485)	38 (7.84)	1.72 (1.11‐2.66)	.02	2.22 (1.25‐3.95)	.007
0.5 (n=498)	21 (4.22)	0.94 (0.56‐1.59)	.83	0.82 (0.38‐1.77)	.61
Condom use in last 6 months (%)			.02		.02
Always (n=982)	45 (4.58)	1 (Reference)		1 (Reference)	
Sometimes or never (n=757)	43 (6.74)	1.65 (1.10‐2.46)		1.88 (1.11‐3.20)	
Sex partners in 6 months (%)					
1-5 (n=1729)	82 (4.74)	1 (Reference)		1 (Reference)	
>5 (n=116)	18 (15.52)	3.54 (2.12‐5.90)	<.001	2.60 (1.28‐5.30)	.008
STI^a^ diagnosis			.005		.15
Ever diagnosed with STI (n=156)	17 (10.90)	1 (Reference)		1 (Reference)	
Never diagnosed with STI (n=1753)	84 (4.79)	0.47 (0.28‐0.79)		0.56 (0.26‐1.24)	
Commercial sexual behavior			.005		.06
Yes (n=107)	12 (11.21)	1 (Reference)		1 (Reference)	
No (n=1801)	89 (4.94)	0.42 (0.23‐0.77)		0.44 (0.18‐1.04)	
Drug contacts			.05		.31
Never contacted with drugs (n=1830)	96 (5.25)	1 (Reference)		1 (Reference)	
Ever contacted with drugs (n=54)	5 (9.26)	2.46 (1.00‐6.07)		1.86 (0.56‐6.18)	

a STI: sexually transmitted infection.

b Not applicable.

**Figure 6.**
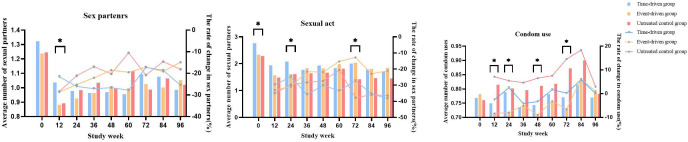
Changes in sexual practice of MSM during the follow-up. MSM: men who have sex with men.

Regarding shifts in sexual behaviors, the number of sexual partners exhibited a fluctuating pattern, initially decreasing across all groups when compared to the baseline, but subsequently increased later during follow-up. Participants in the time-driven group reported the highest number of sexual acts, followed by the event-driven and untreated control groups. However, a positive correlation emerged between condom use and the follow-up period. Notably, participants in the untreated control group reported the highest frequency of condom use, followed by the time-driven and event-driven groups reporting the lowest levels (Figure 6).

The event-driven group exhibits a higher total number of symptom abnormalities (n=33 adverse events) compared to the time-driven group (n=22). Specifically, the number of diarrhea abnormalities was 6 in the time-driven group and 11 in the event-driven group; for vomiting, 1 versus 5; for dizziness, 1 versus 4; and for nausea, 2 versus 3, in the time-driven and event-driven groups, respectively. Conversely, the time-driven group showed a higher count in the "Others" category, with 10 abnormalities compared to 5 in the event-driven group.

## Discussion

### Principal Findings

Although the data from this study are nearly a decade old, our findings remain highly relevant in the field of PrEP, particularly in China. To our knowledge, this study represents the most extensive PrEP trial conducted in China to date, characterized by its comprehensive scope and substantial sample size, which collectively enhance the robustness and generalizability of our findings. Through rigorous statistical analysis, we evaluated the feasibility, efficacy, and safety of TDF as a standalone PrEP intervention. Our analysis demonstrates that while TDF administered as standalone PrEP is safe, it does not provide significant protection against HIV. However, under conditions of high adherence (≥80% dosing compliance), TDF exhibits a substantial 53% reduction in HIV infection risk, underscoring the critical role of medication consistency in achieving prophylactic efficacy. Furthermore, we advocate for an event-driven medication regimen to balance safety with cost-effectiveness. Our analysis also highlights several risk factors for HIV acquisition, including poor PrEP compliance, receptive sexual role, occasional condom use, and having multiple sex partners (>5). Notably, contrary to early studies, we observed sexual behavioral disinhibition during the follow-up period, further underscoring the complexity of sexual practices and the need for targeted interventions [12,26].

The combination of tenofovir and emtricitabine has demonstrated efficacy in preventing HIV acquisition, which was licensed as PrEP in China in August 2020 [12]. However, due to the high incidence of adverse drug reactions observed in pilot trials and the barrier of expensive drug prices, we ultimately decided to explore the efficacy and safety of using TDF alone as PrEP. Initiating treatment with a foundational drug was pivotal in mitigating the potential risk of HIV drug resistance associated with PrEP utilization. While individuals engaging in behaviors associated with heightened HIV risk may experience disparities in certain health indicators, epidemiological data suggest this population often demonstrates baseline physiological resilience. This relative health stability, coupled with tenofovir’s well-documented safety profile in diverse cohorts, supports its consideration as a sustainable PrEP option for long-term use, particularly when integrated with adherence support systems. Furthermore, given the good medication compliance observed in pilot trials and the relatively easier recruitment of individuals, MSM were chosen as participants for the pivotal trials.

The HIV incidence reported in our study was notably lower than that observed in previous investigations [4,12], which may be attributable to the comprehensive health education and consistent condom distribution implemented at every study visit. No statistically significant difference emerged between the medication groups and the untreated control group. However, tenofovir monotherapy was found to confer an additional 53% reduction in the hazard of HIV infection. Furthermore, when medication was administered on an event-driven basis, this protective effect was even more pronounced, with a 62% reduction in the hazard of HIV infection. These findings highlight the importance of adherence and tailored dosing schedules in maximizing the preventative potential of tenofovir as PrEP [27,28].

After comprehensive multivariable adjustment, only poor PrEP compliance was significantly related to sexual practices among the five major hazard components (HR > 2). Adherence to daily PrEP dosing was influenced by several factors, notably diminished motivation when individuals regarded PrEP as a situational preventive tool rather than an integral part of their routine health management. This perception often undermines consistent pill-taking, as prophylactic measures are frequently viewed as temporary responses to risk rather than sustained health practices [29]. Echoing earlier findings from the pioneering PrEP clinical trials and the landmark PROUD study, our results highlight the paramount importance of high medication adherence in maximizing PrEP’s effectiveness in preventing HIV seroconversion [12].

While sexual practices are well-established risk factors for HIV infection [30], our study observed a phenomenon of sexual behavioral disinhibition during the follow-up period. This aligns with a systematic review of 17 open-label PrEP studies, which consistently reported a rise in sexual practice and bacterial STIs among PrEP users [31]. HIV prevention measures encompass HIV testing, the free distribution of condoms, health education, and HIV counseling. After initiating a PrEP scheme, there may be a reduction in condom use and the frequency of HIV testing, or an increase in sexually transmitted infections (STIs). The medication group’s slightly disinhibited behavior could be attributed to a multifaceted interplay of psychological, behavioral, and biological factors. First, risk compensation psychology suggests that individuals may subconsciously adjust their behavior in response to perceived reductions in HIV risk, a cognitive bias where decreased vigilance towards HIV acquisition risks aligns with Prospect Theory—perceived risk reduction increases tolerance for riskier choices [32]. Second, miscalibration of PrEP efficacy may play a role, as overestimation of PrEP’s protective effects (eg, ignoring the need for adherence) or underestimating the persistence of other STIs can fuel riskier practices; notably, a study in JAMA Network Open found that 30% of PrEP users overestimated its efficacy against STIs [13]. Third, social norms and sexual scripting within MSM communities may inadvertently normalize riskier behaviors if narratives around “treatment as prevention” (TasP) or “undetectable=untransmittable” are decoupled from broader sexual health education [31]. Finally, biological factors might contribute, as reduced anxiety about HIV acquisition could heighten sexual arousal and motivation, indirectly elevating behavioral frequency or intensity, even though PrEP itself does not increase libido[加5]. Together, these elements create a complex dynamic that may underpin the observed behavioral disinhibition in the medication group. This indicates the need for comprehensive risk reduction counseling and ongoing education to mitigate potential behavioral changes that may undermine the protective benefits of PrEP. Experience from the USA, UK, and Australia suggests that to address these issues, it is important to enhance STI testing, establish a robust surveillance system, and base prescriptions on both HIV and STI test results [4,6].

Implementing PrEP as a nationwide program is imperative, given the compelling evidence that targeting high-risk individuals with PrEP is not only effective but also cost-efficient [19]. While the adoption of PrEP holds significant promise, it’s crucial to acknowledge a potential drawback: the development of long-term “tolerance,” which could potentially narrow down treatment avenues in the event of an acute HIV infection [33]. As a fundamental antiviral, tenofovir stands out for its ability to circumvent drug resistance concerns that often complicate treatment regimens. Furthermore, its cost-effectiveness is a significant advantage, alleviating financial burdens for both governmental bodies and individual patients alike. By embracing tenofovir-based PrEP, we can forge a path towards a more accessible and sustainable approach to HIV prevention. An event-driven medication regimen was recommended for PrEP. The IPERGAY trial demonstrated that an event-driven regimen of tenofovir disoproxil fumarate-emtricitabine reduced incidence of HIV infection by 86% [34]. Pharmacokinetically, tenofovir reaches its maximum concentration (T_max_) in approximately 1 hour and has a half-life (T_1/2_) of around 18 hours. This event-driven regimen maintains drug concentration at a high level in vivo. Additionally, medication compliance was higher with the event-driven regimen compared to daily dosing. In the event-driven group, the median number of pills used was four per month, compared to an average of sixteen pills reported in the IPERGAY trial for event-driven use, making it significantly less costly than daily medication.

The study has several limitations. First, the absence of a placebo and blinding may introduce bias in the safety assessment, as participants in the time-event group were unwilling to take the medication daily [34]. The use of a placebo or blinding could lead to unblinding, potentially underestimating compliance. Second, to closely mimic real-world conditions, no additional interventions were implemented to maintain the cohort or improve medication compliance during the trial, resulting in a high drop-out rate and poor adherence. The reliance on self-reported adherence and pill counts may also introduce bias, as these methods can be subjective and prone to overestimation. Finally, while the participants in this study may not represent all MSM in China, the large sample size helped mitigate this concern to some extent [35].

### Conclusion

In conclusion, the TDF is ineffective without good adherence. When reliable medication compliance is attainable, event-driven dosing emerges as a highly recommended and effective PrEP strategy. These findings resonate profoundly with the practical challenges and opportunities within China, compellingly underscoring the critical urgency of integrating PrEP into national healthcare policies. By prioritizing and implementing such policies, we can fortify our defenses against the spread of HIV, thereby safeguarding the health and well-being of all citizens and fostering a more resilient public health infrastructure.

## Supplementary material

10.2196/71494 Multimedia Appendix 1 Study questionnaire

10.2196/71494 Checklist 1 CONSORT checklist

## Abbreviations

MSM: men who have sex with men
PrEP: pre-exposure prophylaxis
STI: sexually transmitted infection
TDF: tenofovir disoproxil fumarate
