# Supplementary material for: Tenofovir to Prevent HIV Infection in Western China: Pragmatic Randomized Controlled Trial
Source: JMIR Public Health Surveill. 2025 Aug 20;11:e71494. doi: 10.2196/71494 (PMC12367284; doi:10.2196/71494)
Supplement: Multimedia Appendix 1 [file publichealth-v11-e71494-s001.docx]

**Appendix 1: Questionnaire**

Introduction：

Hello! We are investigators in the feasibility study of Pre-exposure Prophylaxis(PrEP), which is the national major project. In order to understand your cognition of health knowledge, attitude, and behavior, this survey may involve some of your privacy. All of your information will be kept secret, and we promise to only do scientific research and analysis of the population, not personal analysis. So please do the paper according to your true situation. This survey may spare a few minutes, please understand! Thank you very much for your support and cooperation.

**Basic Information**

Questionnaire No：**□□□□□□□□□□**（National standard code of administrative division + category of survey object + number of survey object）

Survey place：___________province（municipality directly under the Central Government）___________city/ district/county

Survey site：____________ Sign the informed consent：_______________（yes/no）

Investigator：____________

Date of interview：date______month______year______

**Form** Questionnaire Quality Supervision Form

| Project | Completion | | Note |
| --- | --- | --- | --- |
|  | yes | no |  |
| The questionnaire is filled in in a standard and clear manner. |  |  |  |
| Every question has been answered without omission |  |  |  |
| No logic error |  |  |  |

Note: Please mark under the corresponding option,“√”

Questionnaire quality supervisor：____________

Quality supervision date：date______month______year______

**Table 1. General information questionnaire**

| **#** | **Question & option** | **Response** | |
| --- | --- | --- | --- |
| A1 | **Ethnicity** |  | |
| A2 | **Year of birth** |  | |
| A3 | **Census register** _________province（municipality directly under the Central Government）_________city/ district/county |  |  |
| A4 | **Household registration：** ①registered urban residents ②registered rural resident |  | |
| A5 | **Current residence**_________province（municipality directly under the Central Government）_________city/ district/county |  |  |
| A6 | **How long have you lived in your current residence：** ①less than 3 months ②3 months to 6 months ③6 months to 1 year ④1 year to 2 years ⑤more than 2 years |  | |
| A7 | **What is the highest level of education you completed?** ①Never attended school & attended school, but never completed any level ②Primary school (grades 1 - 6) ③Secondary school (grades 7 - 9) ④Senior High School (grades 10 - 12) ⑤Vocational training or technician ⑥University or higher |  | |
| A8 | **What is your occupation?** ①Entertainment staff ②General manual workers (including workers, salesmen, apprentices and waiters) ③coach drivers ④students ⑤Government civil servants ⑥institution staff ⑦Employees ⑧migrant workers ⑨Farmers, herdsmen, fishermen (boat) ⑩Self employment (such as self-employed) ⑾Retiree ⑿No income/not employed ⒀Other (specify): |  | |
| A9 | **What is your current marital status?：**①Unmarried, no cohabiting girlfriend ②Unmarried, with a cohabiting girlfriend ③Married ④Married, separated ⑤Married, the spouse is “Lala” (lesbian) ⑥Divorce ⑦Widow ⑧Other (specify): |  | |
| A10 | **Are there any children now：** ①Yes ②No |  | |
| A11 | **If unmarried, will you choose heterosexual marriage in the future：**①Yes ②No ③Do not know |  | |
| A12 | **Current relationship with family:** ①Very good ②Good ③Average ④Poor ⑤Very poor |  | |
| A13 | **What is your overall monthly household disposable income?：__________** ①No income ②Less than 1000 RMB ③1001~3000 RMB ④3001~5000 RMB ⑤5001~7000 RMB ⑥7001~9000 RMB ⑦More than 9000 RMB |  | |
| A14 | **What is the main occupation or activity through which you earn income?**   1. Mainly by myself ②Mainly by spouse/fixed partner ③Mainly by parents ④Other (specify): |  | |
| A15 | **How much do you think you care about your own health?** ① Very concerned ② Concerned ③ Average ④ Not concerned ⑤ Very unconcerned |  | |

**Table 2. AIDS knowledge, attitude and behavior**

| **#** | **Question & option** | | **Response** |
| --- | --- | --- | --- |
| **1、Knowledge & Attitude** | | |  |
| B1 | **Do you know about AIDS?** ①Today is the first time to hear（Skip to D1） ②I’ve heard of it, but I don’t know exactly what it is ③I’ve heard of it, and I also know some relevant knowledge ④Yes, I know this disease better ⑤Yes, I know this disease very well | | （Select①，skip to D1） |
| B2 | **Which of the following ways do you think can spread AIDS?** | |  |
|  | **（1）Importing blood or blood products with AIDS virus** ①Yes ②No ③Do not know | |  |
|  | **（2）Sharing needles with people infected with AIDS**  ①Yes ②No ③Do not know | |  |
|  | **（3）Pregnancy, delivery, and lactation of pregnant infected with AIDS** ①Yes ②No ③Do not know | |  |
|  | **（4）Eating with people infected with AIDS** ①Yes ②No ③Do not know | |  |
|  | **（5）Mosquito bites** ①Yes ②No ③Do not know | |  |
|  | **（6）Having sex with someone who looks clean and healthy** ①Yes ②No ③Do not know | |  |
|  | **（7）Using surgical, acupuncture, tooth extraction, beauty, and other instruments that are not strictly disinfected**  ①Yes ②No ③Do not know | |  |
|  | **（8）Cough and sneeze** ①Yes ②No ③Do not know | |  |
| B3 | **Which of the following ways do you think can prevent AIDS?** | |  |
|  | **（1）Correct use of qualified condoms every time** ①Yes ②No ③Do not know | |  |
|  | **（2）Keeping a sexual partner who is not infected with AIDS** ①Yes ②No ③Do not know | |  |
|  | **（3）Using disposable needles** ①Yes ②No ③Do not know | |  |
|  | **（4）Washing your private parts before and after sex** ①Yes ②No ③Do not know | |  |
|  | **（5）Regular use of anti-AIDS drugs before and after sex** ①Yes ②No ③Do not know | |  |
| B4 | **Is unprotected oral sex possible to infect AIDS?** ①Yes ②No ③Do not know | |  |
| B5 | **Is there any way to cure it now if getting AIDS?** ①Yes ②No ③Do not know | |  |
| B6 | **If a person is infected with AIDS, should she/he be isolated?**   1. Absolutely ②Possibly ③Not very sure ④Irrationally ⑤Not at all | |  |
| B7 | **If a person is infected with AIDS, should he tell his family/spouse?**   1. Absolutely ②Possibly ③Not very sure ④Irrationally ⑤Not at all ⑥Respect personal will | |  |
| B8 | **If your friend is infected with AIDS, what will you do?**   1. Absolutely drop the relationship ②Reduce contacts ③Same as before ④Concern and help ⑤Care to help him | |  |
| B9 | **If your spouse/regular sexual partner is infected with AIDS, do you continue to have sexual relations with her/him?**   1. Absolutely ②Possibly ③Not very sure ④Not possibly ⑤Not at all | |  |
| B10 | **What do you think is the attitude of most people toward those infected with AIDS?**   1. Very discriminatory ②Discriminatory ③Not very sure ④Non-discriminatory ⑤Absolutely non-discriminatory | |  |
| B11 | **What is your attitude towards those infected with AIDS?**  ①Very discriminatory ②Discriminatory ③Not very sure ④Non-discriminatory ⑤Absolutely non-discriminatory | |  |
| B12 | **Are you worried that AIDS will pose a threat to you and your family?**   1. Very concerned ② Concerned ③ Average ④ Not concerned ⑤ Very unconcerned | |  |
| B13 | **How do you think the number of people infected with AIDS now compares with the past?**   1. A great increase ②Increase ③Almost ④Reduced ⑤A great decrease | |  |
| B14 | **How seriously do you think AIDS is?** ① Very serious ② Serious ③ General ④ Not serious ⑤ Not serious at all | |  |
| B15 | **Compared with cancer, how serious do you think AIDS is?**  ①Much more serious than cancer ②More serious than cancer ③Like cancer ④No serious cancer ⑤Far less serious than cancer | |  |
| B16 | **How many people around you do you know are infected with AID?** (If not, fill in “0”, skip to B19) | | (Fill in “0” and skip to B19) |
| B17 | **Do you know how they were infected?**   1. Yes（Please specify ________________） ②No | |  |
| B18 | **Do you know how their sexual behavior has changed since they were infected?** (Multiple choices)   1. Yes, it is crazier than before to find 419 or provide/purchase sexual services ②Yes, no more or less 419 or provide/purchase sexual services ③Yes, other reasons（Please specify ________________） ④Nothing changed | |  |
| B19 | **How many people around you do you know died of AIDS infection?** (If not, fill in “0”) | |  |
| B20 | **Do you think there are any AIDS-infected people/patients in the city/district/county where you live?**   1. A lot ②A few ③None ④Do not know | |  |
| B21 | **Do you think the AIDS epidemic will affect the development of the whole society?**   1. Definitely yes ② Yes ③ Hard to say ④ No ⑤ Definitely not | |  |
| B22 | **Do you think the AIDS epidemic will affect your family life?**  ①Definitely yes ② Yes ③ Hard to say ④ No ⑤ Definitely not | |  |
| B23 | **Do you think the AIDS epidemic will affect you personally?**  ①Definitely yes ② Yes ③ Hard to say ④ No ⑤ Definitely not | |  |
| B24 | **Do you agree with the following statements?** | |  |
|  | **（1）If a person has AIDS, his/her life will be over.**  ① Completely agree ② Basically agree ③ Hard to say ④ Basically disagree ⑤ Completely disagree | |  |
|  | **（2）If a person has AIDS, the whole family will be unlucky with him/her.**  ① Completely agree ② Basically agree ③ Hard to say ④ Basically disagree ⑤ Completely disagree | |  |
|  | **（3）If a person has AIDS, he/she will have no face to others.**  ① Completely agree ② Basically agree ③ Hard to say ④ Basically disagree ⑤ Completely disagree | |  |
| B25 | **Do you think AIDS is far away from your present life?** ① Very far ② Far ③ Hard to say ④ Not far ⑤ Very far | |  |
| B26 | **Do you think the rate of AIDS infection among gay in your city is high?**  ① Very high ② High ③ General ④ Low ⑤ Very low ⑥ Unclear | |  |
| B27 | **Do you think the rate of homosexual netizens on the Internet infected with AIDS is high?**   1. Very high ② High ③ General ④ Low ⑤ Very low ⑥ Unclear | |  |
| **2、Access to the state of AIDS prevention services** | | |  |
| C1 | **Have you received the following services related to AIDS prevention in the past six months?** | |  |
|  | **（1）The release of condoms** ①Yes ②No | |  |
|  | **（2）The release of lubricant** ①Yes ②No | |  |
|  | **（3）Relevant knowledge dissemination among partners** ①Yes ②No | |  |
|  | **（4）AIDS and STD publicity materials (brochures, foldouts, etc.)** ①Yes ②No | |  |
| C2 | **In the past year, have you received free AIDS counseling?** ①Yes (skip to C5) ②No | | (Select ①, skip to C5) |
| C3 | **If you haven’t consulted, would you like to do it?**   1. Very willing (skip to C5) ②Willing (skip to C5) ③Not very sure ④Unwilling ⑤Very unwilling | | (Select ①②, skip to C5) |
| C4 | **If you are unwilling to do it, why?** (Multiple choices)   1. Feel that I am not at risk of contracting AIDS ②Afraid of others knowing that I am consulting ③I don’t know where I can do this consultation ④Embarrassed to do AIDS consultation ⑤Others (______________________________) | |  |
| C5 | **In the last year, have you had an AIDS virus test?**   1. Yes, charged ②Yes, free ③No (skip to C7) | | (Select③, skip to C7) |
| C6 | **In which of the following situations did your test occur?** (Multiple choices) (Skip to C9 after answering this question)  ①I asked for it ②Routine tests, such as conscription, recruitment, welfare physical examination, blood donation ③Examination during medical service (such as before surgery) ④Organized by CDC ⑤Others（_________________） | | (Skip to C9 after answering this question) |
| C7 | **If you haven’t done it, are you willing to do free testing?**   1. Very willing (skip to C9) ②Willing (skip to C9) ③Not very sure ④Unwilling ⑤Very unwilling | | (Select ①、②, skip to C9） |
| C8 | **If not, why not?** (Multiple choices)   1. Feel that I am not at risk of contracting AIDS ②Afraid of others knowing that I do testing ③I don’t know where to do the test ④Embarrassed to do AIDS test ⑤Dare not face it, afraid to detect that I am infected with AIDS ⑥Others(______________) | |  |
| C9 | **What are the main ways for you to obtain knowledge and information about AIDS?** (Multiple choices)   1. Internet ②Television ③Radio broadcast ④Doctor ⑤Relatives ⑥Friends ⑦Newspaper ⑧Books ⑨School education ⑩Publicity materials ⑾Theatrical performances ⑿Advertisement ⒀Consulting service ⒁Drug rehabilitation center/methadone clinic, etc ⒂Peer education in the working group ⒃Do not know ⒄Others（Please specify _______________） ⒅Never obtained | |  |
| C10 | **Are you willing to acquire AIDS prevention knowledge?**   1. Very willing ②Willing ③Not very sure ④Unwilling (skip to C12） ⑤Very unwilling (skip to C12) | | (Select ④、⑤, skip to C12） |
| C11 | **In what ways do you want to get knowledge about AIDS? (**Multiple choices)   1. Internet ②Television ③Radio broadcast ④Doctor ⑤Relatives ⑥Friends ⑦Newspaper ⑧Books ⑨School education ⑩Publicity materials ⑾Theatrical performances ⑿Advertisement ⒀Consulting service ⒁Drug rehabilitation center/methadone clinic, etc ⒂Peer education in the working group ⒃Do not know ⒄Others（Please specify _______________） ⒅Unwilling to obtain | |  |
| C12 | **Have you ever participated in some AIDS prevention public welfare activities or AIDS prevention training conducted by non-governmental organizations (such as gay working groups)? How often?**   1. Frequently ②Occasionally ③Never | |  |
| **3、Behavior Characteristics** | | |  |
| D1 | **Which of the following situations do you think you belong to?：**  ①Homosexuality ②Heterosexuality ③Bisexuality ④Undetermined | |  |
| D2 | **What was your age (one full year of life) when you first had penetrative Sex？** | |  |
| D3 | **Is your first sexual partner male or female?** ①Male（skip to D5） ②female | | (Select ①, skip to D5) |
| D4 | **How old were you when you first had penetrative Sex with a man?** | |  |
| D5 | **How many different men have you had penetrative sex with in the past six months?** | |  |
| D7 | **How you have sex with a male partner：** ①Just as “1” ②Both, mainly “1” ③Both, almost ④Both, mainly “0” ⑤Just as “0” | |  |
| D8 | **How many male fixed sexual partners have you had in the past six months?** (If not, fill “0” and skip to D14) | | （Fill in “0”, skip to D14） |
| D9 | **How often do you use condoms when you have anal sex with a male regular partner in the past six months?**   1. Never used（0%） ②Occasionally used（<20%） ③Used in a few cases（20%~40%） ④Used half the time（40%~60%） ⑤Used most of the time（60%~80%） ⑥Frequently used (>80%) ⑦Every time（100%）（skip to D11） ⑧No anal sex（skip to D12） | | （select ⑦，skip to D11）  （select ⑧，skip to D12） |
| D10 | **Why did you not use a condom when having anal sex with a male fixed partner?** (Multiple choices)   1. I didn’t think about using condoms at that time ②No condom around at that time ③Too expensive ④Not comfortable with condoms ⑤The other party refuses to use ⑥Afraid that the other party thinks I don’t trust him ⑦Embarrassed or dare not ask him to use ⑧Believe that the other person is not ill, so it is unnecessary to use ⑨Afraid of being suspected of having (sexual) disease ⑩Other safety measures have been taken (Please specify _______________) ⑾Others（Please specify____________） | |  |
| D11 | **Did you use a condom when you had anal sex with a male fixed partner last time?** ①Yes ②No | |  |
| D12 | **How often do you use condoms when you have oral sex with a male regular partner in the past six months?**   1. Never used（0%） ②Occasionally used（<20%） ③Used in a few cases（20%~40%） ④Used half the time（40%~60%） ⑤Used most of the time（60%~80%） ⑥Frequently used (>80%) ⑦Every time（100%）（skip to D14 ） ⑧No oral sex（skip to D14） | | (Select ⑦、⑧, skip to D14） |
| D13 | **Why did you not use a condom when having oral sex with a male fixed partner? (Multiple choices)**   1. I didn’t think about using condoms at that time ②No condom around at that time ③Too expensive ④Not comfortable with condoms ⑤The other party refuses to use ⑥Afraid that the other party thinks I don’t trust him ⑦Embarrassed or dare not ask him to use ⑧Believe that the other person is not ill, so it is unnecessary to use ⑨Oral sex does not spread sexually transmitted diseases/AIDS ⑩Other safety measures have been taken (Please specify _______________) ⑾Others（Please specify____________） | |  |
| D14 | **How many male temporary sexual partners have you had in the last six months? (If not, fill “0”, skip to D20)** | | （Fill “0”, skip to D20） |
| D15 | **Why did you not use a condom when having anal sex with a male temporary partner?**   1. Never used（0%）②Occasionally used（<20%） ③Used in a few cases（20%~40%）④Used half the time（40%~60%） ⑤Used most of the time（60%~80%） ⑥Frequently used (>80%) ⑦Every time（100%）（skip to D17） ⑧No anal sex（skip to D18） | | （Select⑦, skip to D17）  （Select⑧, skip to D18） |
| D16 | **Why did you not use a condom when having anal sex with a male temporary partner? (Multiple choices)**   1. I didn’t think about using condoms at that time ②No condom around at that time ③Too expensive ④Not comfortable with condoms ⑤The other party refuses to use ⑥Afraid that the other party thinks I don’t trust him ⑦Embarrassed or dare not ask him to use ⑧Believe that the other person is not ill, so it is unnecessary to use ⑨Afraid of being suspected of having (sexual) disease ⑩Other safety measures have been taken (Please specify _______________) ⑾Others（Please specify____________） | |  |
| D17 | **Did you use a condom when you had anal sex with a male fixed partner last time?** ①Yes ②No | |  |
| D18 | **How often do you use condoms when you have oral sex with a male temporary partner in the past six months?**   1. Never used（0%） ②Occasionally used（<20%） ③Used in a few cases（20%~40%） ④Used half the time（40%~60%） ⑤Used most of the time（60%~80%） ⑥Frequently used (>80%) ⑦Every time（100%）（skip to D20 ） ⑧No oral sex（skip to D20） | | （Select⑦、⑧，skip to D20） |
| D19 | **Why did you not use a condom when having oral sex with a male temporary partner? (Multiple choices)**   1. I didn’t think about using condoms at that time ②No condom around at that time ③Too expensive ④Not comfortable with condoms ⑤The other party refuses to use ⑥Afraid that the other party thinks I don’t trust him ⑦Embarrassed or dare not ask him to use ⑧Believe that the other person is not ill, so it is unnecessary to use ⑨Oral sex does not spread sexually transmitted diseases/AIDS ⑩Other safety measures have been taken (Please specify _______________) ⑾Others（Please specify____________） | |  |
| D20 | **In the past six months, what are the main ways for you to find a sexual partner:** (Multiple choices)   1. Gay bar ②Dance halls, teahouses and clubs ③Bath, sauna, foot massage ④In parks, public toilets and green belts ⑤Internet connection ⑥Introduced by friends ⑦Purchasing services ⑧Provision of sexual services ⑨Others（Please specify____________） | |  |
| D21 | **How often do you search for sexual partners through the Internet？**   1. Frequently ②Sometimes ③Occasionally ④Never | |  |
| D22 | **In the past six months, have you ever received sexual services from men by paying for them?** ①Yes ②No（skip to D29） | | （Select ②, skip to D29） |
| D23 | **In the past six months, how many men have provided you with sexual services by paying for them?** | |  |
| D24 | **How often do you use condoms when having anal sex with these people in the past six months?**   1. Never used（0%） ②Occasionally used（<20%） ③Used in a few cases（20%~40%） ④Used half the time（40%~60%） ⑤Used most of the time（60%~80%） ⑥Frequently used (>80%) ⑦Every time（100%）（skip to D26） ⑧No anal sex（skip to D27） | | （Select⑦, skip to D26）  （Select⑧, skip to D27） |
| D25 | **Why did you not use a condom when having anal sex with these men?** (Multiple choices)   1. I didn’t think about using condoms at that time ②No condom around at that time ③Too expensive ④Not comfortable with condoms ⑤The other party refuses to use ⑥Afraid that the other party thinks I don’t trust him ⑦Embarrassed or dare not ask him to use ⑧Believe that the other person is not ill, so it is unnecessary to use ⑨Afraid of being suspected of having (sexual) disease ⑩Other safety measures have been taken (Please specify _______________) ⑾Others（Please specify____________） | |  |
| D26 | **Did you use a condom when you last had anal sex with these people?** ①Yes ②No | |  |
| D27 | **How often do you use condoms during oral sex with these people in the last six months?**   1. Never used（0%） ②Occasionally used（<20%） ③Used in a few cases（20%~40%） ④Used half the time（40%~60%） ⑤Used most of the time (60%~80%) ⑥Frequently used (>80%) ⑦Every time（100%）（skip to D29） ⑧No oral sex (skip to D29) | | (Select ⑦、⑧，skip to D29) |
| D28 | **Why did you not use a condom when having oral sex with a male temporary partner? (**Multiple choices)   1. I didn’t think about using condoms at that time ②No condom around at that time ③Too expensive ④Not comfortable with condoms ⑤The other party refuses to use ⑥Afraid that the other party thinks I don’t trust him ⑦Embarrassed or dare not ask him to use ⑧Believe that the other person is not ill, so it is unnecessary to use ⑨Oral sex does not spread sexually transmitted diseases/AIDS ⑩Other safety measures have been taken (Please specify _______________) ⑾Others（Please specify____________） | |  |
| D29 | **In the past six months, have you provided commercial sexual services for men in order to get money?**  ①Yes ②No（skip to D36） | | （Select ②，skip to D36） |
| D30 | **In the past six months, how many men have you provided commercial sexual services for money?** | |  |
| D31 | **How often do you use condoms when having anal sex with these men in the past six months?**   1. Never used（0%） ②Occasionally used（<20%） ③Used in a few cases（20%~40%） ④Used half the time（40%~60%） ⑤Used most of the time（60%~80%） ⑥Frequently used (>80%) ⑦Every time（100%）（skip to D33） ⑧No anal sex（skip to D34） | | （Select ⑦，skip to D33）  （Select ⑧，skip to D34） |
| D32 | **Why did you not use a condom when having anal sex with these men? (**Multiple choices)   1. I use condoms every time ② I didn’t think about using condoms at that time ③No condom around at that time ④Too expensive ⑤Not comfortable with condoms ⑥The other party refuses to use ⑦The guest paid me more for not using a condom ⑧Afraid that the other party thinks I don’t trust him ⑨The guest suspects that I have (sexual) disease ⑩Embarrassed or dare not ask him to use ⑾The other party doesn’t seem to be ill, so it's unnecessary ⑿Other safety measures have been taken (Please specify _______________) ⒀Others（Please specify____________） | |  |
| D33 | **Did you use a condom when you last had anal sex with these men?** ①Yes ②No | |  |
| D34 | **How often do you use condoms during oral sex with these people in the last six months?**   1. Never used（0%） ②Occasionally used（<20%） ③Used in a few cases（20%~40%） ④Used half the time（40%~60%） ⑤Used most of the time (60%~80%) ⑥Frequently used (>80%) ⑦Every time（100%）（skip to D36） ⑧No oral sex (skip to D36) | | (Select ⑦⑧, skip to D36) |
| D35 | **Why did you not use a condom when having oral sex with these men? (Multiple choices)**   1. I didn’t think about using condoms at that time ②No condom around at that time ③Too expensive ④Not comfortable with condoms ⑤The other party refuses to use ⑥The guest paid me more for not using a condom ⑦Afraid that the other party thinks I don’t trust him ⑧Embarrassed or dare not ask him to use ⑨The other party doesn’t seem to be ill, so it’s unnecessary ⑩Oral sex does not spread sexually transmitted diseases/AIDS ⑾Other safety measures have been taken (Please specify _______________) ⑿Others（Please specify____________） | |  |
| D36 | **Have you had sex with women in the last six months?** ①Yes ②No（skip to D43） | | (Select ②, skip to D43) |
| D37 | **How many different women have you had sex with in the past six months?** | |  |
| D38 | **How often do you use condoms when you have sex with women (vaginal or anal) in the past six months?**   1. Never used（0%） ②Occasionally used（<20%） ③Used in a few cases（20%~40%） ④Used half the time（40%~60%） ⑤Used most of the time (60%~80%) ⑥Frequently used (>80%) ⑦Every time（100%）（skip to D40） ⑧No vaginal or anal sex（skip to D41） | | (Select ⑦, skip to D40)  (Select ⑧, skip to D41) |
| D39 | **Why did you not use a condom when you had sex with a woman (vaginal or anal)?**（Multiple choices）  ①I didn’t think about using condoms at that time ②No condom around at that time ③Too expensive ④Not comfortable with condoms ⑤The other party refuses to use ⑥Afraid that the other party thinks I don’t trust him ⑦Embarrassed or dare not ask him to use ⑧Believe that the other person is not ill, so it is unnecessary to use ⑨Afraid of being suspected of having (sexual) disease ⑩Other contraceptive measures ⑾Plan to have a baby ⑿Other safety measures have been taken (Please specify _______________) ⒀Others（Please specify____________） | |  |
| D40 | **Did you use a condom during your last sexual intercourse (vaginal or anal) with a woman?** ①Yes ②No | |  |
| D41 | **How often do you use condoms during oral sex with women in the last six months?**   1. Never used（0%） ②Occasionally used（<20%） ③Used in a few cases（20%~40%） ④Used half the time（40%~60%） ⑤Used most of the time (60%~80%) ⑥Frequently used (>80%) ⑦Every time（100%）（skip to D43） ⑧No oral sex（skip to D43） | | (Select ⑦、⑧, skip to D43) |
| D42 | **Why did you not use a condom when you had oral sex with a woman? (Multiple choices)**   1. I didn’t think about using condoms at that time ②No condom around at that time ③Too expensive ④Not comfortable with condoms ⑤The other party refuses to use ⑥Afraid that the other party thinks I don’t trust him ⑦Embarrassed or dare not ask him to use ⑧Believe that the other person is not ill, so it is unnecessary to use ⑩Oral sex does not spread sexually transmitted diseases/AIDS ⑾Other safety measures have been taken (Please specify _______________) ⑿Others（Please specify____________） | |  |
| D43 | **Do you use condoms all the time when you have sex with a male/female partner?**   1. Not used throughout each time（0%） ②Occasionally used throughout（<20%） ③Used throughout in a small part of the time（20%~40%） ④Used throughout in half cases（40%~60%） ⑤Used throughout most of the time（60%~80%） ⑥Always used throughout(>80%) ⑦Used throughout each time（100%） | |  |
| D44 | **Whether the people you know who have had sexual relations with you (male/female) are now found to be AIDS positive.**   1. Yes ②No ③Not very sure | |  |
| D45 | **Have you ever had the following symptoms? (**Urination pain or burning sensation; Abnormal urethral secretion (male)/abnormal vaginal secretion (female); Genitalia has skin damage, ulcer or puffiness; Sexual intercourse pain; Severe or persistent pelvic pain)   1. Yes ②No（skip to D47） | | (Select ②, skip to D47) |
| D46 | **How to solve (venereal disease) symptoms generally?**   1. Go to STD clinic ②In a general hospital at or above the county level ③Go to the Maternity and Child Health Hospital ④Visit a private clinic ⑤Township health center (community health service center) ⑥Village clinic (community health service station) ⑦Buy medicine myself ⑧Not treated ⑨Others（Please specify____________） | |  |
| D47 | **Have you ever been diagnosed with venereal diseases by a doctor?**   1. Yes ②No（Skip to D50） | | (Select ②, skip to D50） |
| D48 | **What disease did the doctor tell you?** （Multiple choices）   1. Syphilis ②Genital herpes ③Gonorrhea ④Condyloma acuminatum ⑤Nonspecific urethritis ⑥Others (Please specify____________) ⑦Unclear |  | |
| D49 | **When was the last sexually transmitted disease?** ①Within one year ②One year ago ③Three years ago ④Five years ago |  | |
| D50 | **How often do you drink in the last six months?**   1. Basically every day ②At least 3 times/week ③At least 1 times/week ④Less than once a week ⑤Never drink (skip to D52) | | (Select ⑤, skip to D52) |
| D51 | **How did you use alcohol before having sex with your partner?**  ①Never used（0%） ②Occasionally used（<20%） ③Used in a few cases（20%~40%） ④Used half the time（40%~60%） ⑤Used most of the time (60%~80%) ⑥Frequently used (>80%) ⑦Every time（100%） | |  |
| D52 | **Which of the following substances have you used in the past six months? (Multiple choices)**   1. None (skip to D57) ②Ecstasy ③Ice ④Ketamine ⑤Opium ⑥Hemp ⑦Heroin ⑧Ma gu (methamphetamine) pill ⑨Dolantin ⑽ Morphine ⑾Others (Please specify____________) | | (Select ①, skip to D57) |
| D53 | **Have you used the above substances before sex in the last six months?**   1. Yes ②No（skip to D55） | | (Select ②, skip to D55） |
| D54 | **How about taking the above drugs before you have sex with your partner in the past six months?**  ①Never used（0%） ②Occasionally used（<20%） ③Used in a few cases（20%~40%） ④Used half the time（40%~60%） ⑤Used most of the time (60%~80%) ⑥Frequently used (>80%) ⑦Every time（100%） | |  |
| D55 | **Have you injected the above substances in the past six months？** ①Yes ②No（skip to D57） | | (Select ②, skip to D57) |
| D56 | **Do you know whether those who have shared needles with you have been found to be AIDS-positive?**   1. Yes ②No ③Unclear ④Not shared | |  |
| D57 | **How likely do you think you are to be infected with AIDS?**  ①Very large ② Large ③ General ④ Small ⑤ Very small |  | |
| D58 | **You think your risk of AIDS mainly comes from:_______ (Multiple choices and sorting)**   1. Male fixed sexual partner ②Male temporary sexual partner ③Male sex service providers ④Male guests ⑤Female ⑥Others (Please specify____________) |  | |

**Table 3. Knowledge, attitude, and willingness to use AIDS prevention measures**

| **#** | **Question & option** | | | | **Response** | |
| --- | --- | --- | --- | --- | --- | --- |
| E1 | **Do you think it is difficult to prevent AIDS at present?**  ① Very difficult ② Difficult ③ General ④ Not difficult ⑤ Not at all | | | |  | |
| E2 | **Do you think you have the ability to prevent AIDS infection?**   1. Fully capable ②Capable ③Hard to say ④Incapable ⑤Totally incapable | | | |  | |
| E3 | **Do you agree with the following statements?** | | | |  | |
|  | 1. **Few men like to use condoms**   ①Completely agree ② Basically agree ③ Hard to say ④ Basically disagree ⑤ Completely disagree | | | |  | |
|  | 1. **If the partner does not ask, I will take the initiative to use condoms** 2. Completely agree ② Basically agree ③ Hard to say ④ Basically disagree ⑤ Completely disagree | | | |  | |
|  | 1. **If you insist on using condoms, others will think you have STDs** 2. Completely agree ② Basically agree ③ Hard to say ④ Basically disagree ⑤ Completely disagree | | | |  | |
|  | 1. **If the other person is unwilling to use a condom, you can persuade him to use a condom** 2. Completely agree ② Basically agree ③ Hard to say ④ Basically disagree ⑤ Completely disagree | | | |  | |
|  | 1. **If the other person refuses to use a condom, you will refuse to have sex with him** 2. Completely agree ② Basically agree ③ Hard to say ④ Basically disagree ⑤ Completely disagree | | | |  | |
|  | 1. **You know how to use condoms correctly** 2. Completely agree ② Basically agree ③ Hard to say ④ Basically disagree ⑤ Completely disagree | | | |  | |
|  | 1. **If you insist on using condoms, the other person will be angry** 2. Completely agree ② Basically agree ③ Hard to say ④ Basically disagree ⑤ Completely disagree | | | |  | |
|  | 1. **Save time and money without condoms** 2. Completely agree ② Basically agree ③ Hard to say ④ Basically disagree ⑤ Completely disagree | | | |  | |
|  | 1. **Using condoms can protect oneself from sexually transmitted diseases and AIDS** 2. Completely agree ② Basically agree ③ Hard to say ④ Basically disagree ⑤ Completely disagree | | | |  | |
| E4 | **Have you heard of the following measures to prevent AIDS before? What do you think its possible preventive effect is (the investigator explains the following measures)** | | | |  | |
|  | **（1）Vaginal/rectal microbicide**  ①Heard ②Never heard | | | |  | |
|  | **You think its possible preventive effect**：① Very good ② Good ③ Average ④ Poor ⑤ Very poor | | | |  | |
|  | **（2）Drug use after AIDS exposure** ①Heard ②Never heard | | | |  | |
|  | **You think its possible preventive effect：**① Very good ② Good ③ Average ④ Poor ⑤ Very poor | | | |  | |
|  | **（3）Pre-exposure medication for AIDS** ①Heard ②Never heard | | | |  | |
|  | **You think its possible preventive effect：**① Very good ② Good ③ Average ④ Poor ⑤ Very poor | | | |  | |
| E5 | **Have you ever used drugs to prevent sexually transmitted diseases before or after high-risk behaviors?**   1. Used ②Unused（skip to E10） | | | | (Select ②, skip to E10) | |
| E6 | **If you have used such medicine, which one?** | | | |  | |
| E7 | **The usage of drugs is：** ①Oral ②External ③Injection | | | |  | |
| E8 | **Who suggested/gave you use? (**Multiple choices)   1. Doctor ②Sexual partner ③Friend ④Others (Please specify____________) ⑤No one, I used it myself | | | |  | |
| E9 | **The specific usage time is: (**Multiple choices)   1. Before the occurrence of high-risk behaviors ②After high-risk behavior ③Regular use during (multiple) high-risk behaviors | | | |  | |
| E10 | **Have you ever used drugs to prevent AIDS before or after high-risk behaviors?** ①Used ②Never used （Skip to E15） | | | | (Select ②, skip to E15) | |
| E11 | **If you have used such medicine, which one?** | | | |  | |
| E12 | **The usage of drugs is：** ①Oral ②External ③Injection | | | |  | |
| E13 | **Who suggested/gave you use? (**Multiple choices)  ①Doctor ②Sexual partner ③Friend ④Others (Please specify____________) ⑤No one, I used it myself | | | |  | |
| E14 | **The specific usage time is:** (Multiple choices)  ①Before the occurrence of high-risk behaviors ②After high-risk behavior ③Regular use during (multiple) high-risk behaviors | | | |  | |
| E15 | **Do you know anyone around you who has used drugs to prevent AIDS?** ①Yes ②No（skip to E19） | | | | (Select ②, skip to E19) | |
| E16 | **If someone around you has used such medicine, which one?** | | | |  | |
| E17 | **The usage of drugs is：** ①Oral ②External ③Injection | | | |  | |
| E18 | **The specific usage time is:** (Multiple choices)  ①Before the occurrence of high-risk behaviors ②After high-risk behavior ③Regular use during (multiple) high-risk behaviors | | | |  | |
| E19 | **If you do not take preventive measures when you have sex with a male fixed partner, what do you think your chances of contracting AIDS are?**  ①Very large ② Large ③ General ④ Small ⑤ Very small | | | |  | |
| E20 | **If you do not take preventive measures when having sex with a temporary male partner, what do you think your chances of contracting AIDS are?**  ①Very large ② Large ③ General ④ Small ⑤ Very small | | | |  | |
| E21 | **If you don't take preventive measures when having sex with a heterosexual partner, what do you think your chances of getting AIDS are?**  ①Very large ② Large ③ General ④ Small ⑤ Very small | | | |  | |
| E22 | **If the rate of AIDS infection among gay men in your city is very high, what measures would you take to prevent AIDS?**   1. Keep a regular partner and stop looking for a temporary partner ②Still look for temporary partners and insist on using condoms every time ③I don’t care. I’m not so susceptible to AIDS ④Others (Please specify____________) | | | |  | |
| E23 | **If it is proved that drug use before AIDS exposure is safe and effective to prevent AIDS, do you think whether this drug should be popularized among gay?**   1. Absolutely should ②Should ③Hard to say ④Should not ⑤Not at all | | | |  | |
| E24 | **Here is the test about your intention to use drugs before AIDS exposure** | | | |  | |
|  | **（1）If medication before AIDS exposure is safe and effective for AIDS prevention, will you use it?**  ①Definitely (skip to E25) ② Probably ③ Not very sure ④ Probably not ⑤ Definitely not | | | | (Select ①, skip to E25) | |
|  | **（2）If medication before AIDS exposure is safe, effective and free for AIDS prevention, will you use it?**  ①Definitely (skip to E25) ② Probably ③ Not very sure ④ Probably not ⑤ Definitely not | | | | (Select ①, skip to E25) | |
| ) | **（3）If medication before AIDS exposure is safe, effective and free for AIDS prevention, and a few people around you are taking this drug, will you use it？**①Definitely (skip to E25) ② Probably ③ Not very sure ④ Probably not ⑤ Definitely not | | | | (Select ①, skip to E25) | |
|  | **（4）If medication before AIDS exposure is safe, effective and free for AIDS prevention, and there are many people around you taking this drug, will you use it?**  ①Definitely (skip to E25) ② Probably ③ Not very sure ④ Probably not ⑤ Definitely not | | | | (Select ①, skip to E25) | |
| E25 | **If medication before AIDS exposure is safe and effective for AIDS prevention, would you recommend it to your friends?**  ①Definitely ② Probably ③ Not very sure ④ Probably not ⑤ Definitely not | | | |  | |
| E26 | **What are your concerns about the medication before AIDS exposure? Please sort according to your most concerned aspects. (**Multiple choices and sorting)   1. Effect ②Safety (side effects) ③Cost ④Accessibility ⑤Convenience of taking medicine ⑥Opinions of people around ⑦Support from sexual partners ⑧Family support ⑨Whether it is used by people around ⑩Others (Please specify____________) ⑾I don’t care | | | |  | |
| E27 | **Are you worried about being discovered by others while taking medicine? Who? (**Multiple choices)   1. No, do not worry about being found ②Male fixed sexual partner ③Male temporary sexual partner ④Spouse/girlfriend ⑤Other family members ⑥Friends ⑦Others | | | |  | |
|  |  | Spouse/girlfriend | Male fixed | Male temporary | Other family members | Friend |
| E28 | **What do you think the following people’s attitude towards your medication?**   1. Support ②Unclear ③Object ④Without such relationship |  |  |  |  |  |
|  |  | (Select ④，skip to the next one) (Select ④, skip to the next one） | | | | |
| E29 | **Do you think the attitude of the following people will affect your choice?**   1. Definitely ②Probably ③Unclear ④Probably not ⑤Definitely not |  |  |  |  |  |
| E30 | **Do you agree with the following statements?** | | | |  | |
|  | 1. **If others know that you are taking this drug, they will discriminate against you** 2. Completely agree ② Basically agree ③ Hard to say ④ Basically disagree ⑤ Completely disagree | | | |  | |
|  | 1. **No matter what other people think, I believe that I can persist in taking this medicine**   ①Completely agree ② Basically agree ③ Hard to say ④ Basically disagree ⑤ Completely disagree | | | |  | |
| **If you are definitely unwilling to take medication before AIDS exposure, end the questionnaire** | | | | |  | |
| E31 | **If you need to spend money on medication before AIDS exposure, how much would you like to spend every month at most?**   1. Unwilling to spend money ②<100 RMB ③100～RMB ④200～RMB ⑤400～RMB ⑥600～RMB ⑦800～RMB ⑧>1000 RMB | | | |  | |
| E32 | **If you need to take this medicine every day, will you insist on taking it?**   1. Definitely (skip to E34) ② Probably (skip to E34) ③ Not very sure ④ Probably not ⑤ Definitely not | | | | (Select ①、②, skip to E34) | |
| E33 | **If you do not want to take it every day, the shortest period you can endure is:**  ①Once every other day ②Once every three days ③Once a week ④Once every half month ⑤Once a month ⑥Others (Please specify____________) | | | |  | |
| E34 | **If there are two types of drugs, the first one is cheap, but the interval is short. The second one is expensive, but the interval is long. Which one do you prefer?** ①The first ②The second ③Unclear | | | |  | |
| E35 | **Which way do you want to know the information about medication before AIDS exposure?**（Multiple choices）   1. Internet ②Television ③Radio broadcast ④Doctor ⑤Relatives ⑥Friends ⑦Newspaper ⑧Books ⑨School education ⑩Publicity materials ⑾Theatrical performances ⑿Advertisement ⒀Consulting service ⒁Drug rehabilitation center/methadone clinic, etc ⒂Peer education in the working group ⒃Do not know ⒄Others（Please specify _______________） ⒅Unwilling to obtain | | | |  | |
| E36 | **Where do you want to get pre-exposure medication for AIDS?**（Multiple choices）  ①Village clinic/health service station ②Township Health Center/Health Service Center ③Hospitals above county (district) level ④Centers for Disease Control and Prevention ⑤AIDS advisory body ⑥STD Specialist Clinic ⑦Detoxification center/methadone clinic ⑧Private clinic ⑨Pharmacy ⑩Adult Health Products Store ⑾Others（Please specify _______________） | | | |  | |
| E37 | **The reason why you choose to obtain pre-exposure medication for AIDS in this location is：**（Multiple choices）  ①Strong confidentiality ②Convenience ③The organization is reliable ④Others（Please specify _______________） | | | |  | |
| E38 | **If you use pre-exposure medication, how often do you think you will use condoms when having sex with others?**   1. Definitely increase ②Probably increase ③Same as before ④Probably descend ⑤ Definitely descend | | | |  | |
| E39 | **If you use pre-exposure medication, what do you think about the number of your sexual partners?**  ①Definitely increase ②Probably increase ③Same as before ④Probably descend ⑤ Definitely descend | | | |  | |
| E40 | **Are you willing to participate in the clinical trial of drug use before AIDS exposure?**  ① Very willing ② Willing ③ Hard to say ④ Not willing (skip to E42) ⑤ Very unwilling (skip to E42) | | | | (Select ④、⑤, skip to E42） | |
| E41 | **The reason why you (may) want to participate in the AIDS pre-exposure drug test is：**（Multiple choices）   1. Worried about the risk of AIDS ②Hope to promote a preventive medication before AIDS exposure ③Participate in the trial to take preventive drugs free of charge ④Others（Please specify _______________） | | | |  | |
| E42 | **The reason why you (probably) do not want to participate in the drug test before AIDS exposure is：**（Multiple choices）   1. They are not in danger of suffering from AIDS, so it is unnecessary to participate ②Worry about side effects of drugs ③Worry that the medicine had no effect ④Worry about being discriminated against by others when taking drugs ⑤Worry about sexual partners’ opposition ⑥Worry about family opposition ⑦Others（Please specify _______________） | | | |  | |
